# PASSED: Brain atrophy in non-demented individuals in a long-term longitudinal study from two independent cohorts

**DOI:** 10.3389/fnagi.2023.1121500

**Published:** 2023-02-22

**Authors:** Anna-Lena Haas, Pauline Olm, Janine Utz, Eva-Maria Siegmann, Philipp Spitzer, Anna Florvaag, Manuel Alexander Schmidt, Arnd Doerfler, Piotr Lewczuk, Johannes Kornhuber, Juan Manuel Maler, Timo Jan Oberstein, for the Alzheimer’s Disease Neuroimaging Initiative

**Affiliations:** ^1^Department of Psychiatry and Psychotherapy, Friedrich-Alexander-Universität Erlangen-Nürnberg, Erlangen, Germany; ^2^Institute of Neuroradiology, Friedrich-Alexander-Universität Erlangen-Nürnberg, Erlangen, Germany; ^3^Department of Neurodegeneration Diagnostics, Department of Biochemical Diagnostics, University Hospital of Bialystok, Medical University of Bialystok, Bialystok, Poland

**Keywords:** magnetic resonance imaging, medial temporal lobe, tau proteins/cerebrospinal fluid*, amyloid beta-peptides/cerebrospinal fluid*, SNAP, ATN classification, Alzheimer disease/physiopathology*, longitudinal studies

## Abstract

**Introduction:**

Alzheimer’s disease (AD) is indicated by a decrease in amyloid beta 42 (Aβ42) level or the Aβ42/Aβ40 ratio, and by increased levels of Tau with phosphorylated threonine at position 181 (pTau181) in cerebrospinal fluid (CSF) years before the onset of clinical symptoms. However, once only pTau181 is increased, cognitive decline in individuals with subjective or mild cognitive impairment is slowed compared to individuals with AD. Instead of a decrease in Aβ42 levels, an increase in Aβ42 was observed in these individuals, leading to the proposal to refer to them as nondemented subjects with increased pTau-levels and Aβ surge with subtle cognitive deterioration (PASSED). In this study, we determined the longitudinal atrophy rates of AD, PASSED, and Biomarker-negative nondemented individuals of two independent cohorts to determine whether these groups can be distinguished by their longitudinal atrophy patterns or rates.

**Methods:**

Depending on their CSF-levels of pTau 181 (T), total Tau (tTau, N), Aβ42 or ratio of Aβ42/Aβ40 (A), 185 non-demented subjects from the Alzheimer’s Disease Neuroimaging Initiative (ADNI) and 62 non-demented subjects from Erlangen AD cohort were assigned to an ATN group (A–T–N–, A–T+N±, A+T–N±and A+T+N±) and underwent T1-weighted structural magnetic resonance imaging (sMRI). Longitudinal grey matter (GM) atrophy patterns were assessed with voxel-based morphometry (VBM) using the cat12 toolbox on spm12 (statistical parametric mapping) of MRI scans from individuals in the ADNI cohort with a mean follow-up of 2 and 5 years, respectively. The annualized atrophy rate for individuals in the Erlangen cohort was determined using region of interest analysis (ROI) in terms of a confirmatory analysis.

**Results:**

In the A–T+N± group, VBM did not identify any brain region that showed greater longitudinal atrophy than the A+T+N±, A+T+N± or biomarker negative control group. In contrast, marked longitudinal atrophy in the temporal lobe was evident in the A+T–N± group compared with A+T–N±  and biomarker-negative subjects. The ROI in the angular gyrus identified by VBM analysis of the ADNI cohort did not discriminate better than the hippocampal volume and atrophy rate between AD and PASSED in the confirmatory analysis.

**Discussion:**

In this study, nondemented subjects with PASSED did not show a unique longitudinal atrophy pattern in comparison to nondemented subjects with AD. The nonsignificant atrophy rate compared with controls suggests that increased pTau181-levels without concomitant amyloidopathy did not indicate a neurodegenerative disorder.

## 1. Introduction

The most common neurodegenerative disorder is Alzheimer’s disease with amyloid plaques and neurofibrillary tangles as neuropathological hallmarks. A biological definition of AD proposes to include surrogate markers for amyloidopathy (A), neurofibrillary tangles (T) and neurodegeneration (N) (Jack et al., 2018a). Surrogate markers for neurodegeneration (N) at the onset of Alzheimer’s disease are atrophy of the mesial temporal lobe, in particular the hippocampal formation, and an increase of total Tau in cerebrospinal fluid (CSF; Jack et al., 2018a). For amyloid plaques, the surrogate markers are a decrease of Aβ42 or a lowered level of the Aβ42/Aβ40 ratio in CSF (McKhann et al., 2011; Dubois et al., 2014; Hansson et al., 2019). For neurofibrillary tangles (T), the surrogate marker can be a higher level auf pTau181 in CSF (Blennow et al., 1995; Hampel et al., 2004; McKhann et al., 2011; Dubois et al., 2016; Jack et al., 2018a). Further, both plaques and tangles can be detected by positron emission tomography (PET) using Aβ or pTau binding tracers (McKhann et al., 2011; Dubois et al., 2016; Jack et al., 2018a). As long as both A and T are pathologically altered, i.e., A+T+, the presence of Alzheimer’s disease is likely (Jack et al., 2018a). In many cases, however, A and T are not congruently changed; for example, the A–T+ group alone comprises up to 23% of cases in cohorts of non-demented elderly (Jack et al., 2018a). Together with the group with evidence of neurodegeneration without amyloidopathy (A–T–N+), it was proposed that they be collectively referred to as suspected non-AD pathophysiology (SNAP) because nondemented individuals with these biomarker constellations had no or only slightly different cognitive trajectories and longitudinal hippocampal atrophy rates compared with control subjects (Burnham et al., 2016; Jack et al., 2018a; Oberstein et al., 2022). The classification of individuals with elevated pTau without amyloidopathy remains controversial, as pTau181 elevation was reported to be specific in AD at least compared with frontotemporal dementia and lewy body dementia and the recently reported specificity of elevation of pTau181 in AD patients in serum (Blennow et al., 1995; Hansson et al., 2019; Thijssen et al., 2020). Moreover, although this biomarker constellation does not seem to be necessarily associated with AD, it was associated with Aβ, as Aβ42 and Aβ40 levels were concomitantly increased with pTau181 in this group, so we proposed to refer to the biomarker constellation in non-demented individuals as PASSED, a **p**Tau and **A**β **s**urge with **s**ubtl**e d**eterioration (Oberstein et al., 2022). The strong association between pTau181 and tTau in PASSED and A–T+ individuals, respectively, indicates the presence of neurodegeneration by definition, but is not reflected in increased longitudinal atrophy of the mesial temporal lobe and hippocampus, respectively. (Burnham et al., 2016) To determine whether neurodegeneration in the sense of longitudinal atrophy occurs in other brain regions, we used voxel-based morphometry (VBM) in this study to compare the distribution of longitudinal GM volume loss of non-demented A–T + individuals to those with A + T– and A + T + and CSF-biomarker negative subjects. The identified brain regions were tested in a confirmatory analysis using ROI analysis in a second independent cohort.

## 2. Materials and methods

### 2.1. Study population

We selected 185 subjects from the Alzheimer’s Disease Neuroimaging Initiative (ADNI).^1^ The ADNI is a longitudinal multicenter study founded 2004 by Principal Investigator M. Weiner as a public-private partnership, and enrolls participants from all over North America. With the data of various imaging and clinical assessments and their sharing of the data for researchers worldwide, ADNI aims at improving diagnosing and treating of AD. Further information about the ADNI cohort, the study protocol and MR image acquisition and processing can be accessed *via*.^2^ This study included only participants who were over 50 years of age and had an analysis of Aβ42 and pTau181 levels in CSF, a neuropsychological assessment with a Mini Mental State Examination (MMSE) score greater than 23, and a structural brain examination with a magnetization-prepared rapid acquisition gradient echo (MP-RAGE) sequence at baseline and at least 12 months later. The biomarker negative control group (Aβ42–T–N–) also had to have normal tTau values to be included in this study. Subjects with focal brain lesions or defects on MRI or significant T2w white matter hyperintensities, i.e., Fazekas 2 or 3, were excluded after inspection of the native and segmented images (Fazekas et al., 1987; Oberstein et al., 2022). 2,150 individuals from the ADNI were screened for eligibility, of which 1,250 had CSF diagnostics with the required parameters and of those, 381 had an MMSE greater than 23. One hundred and ninety four of these had at least one additional MRI > 12 months after the baseline examination of which 9 were excluded due to poor image quality or processing issues. The study population included data from ADNI 1, ADNI 2, ADNIGO, and ADNI3.

From the Erlangen cohort, 62 individuals over 50 years of age with MCI or SCI were included in this study from April 2010 to November 2021. Inclusion in the study was contingent on the presence of a complete set of CSF parameters (Aβ42/Aβ40 ratio, Aβ42-, pTau181-and tTau-level), a neuropsychological assessment with the German version of the CERAD neuropsychological battery, a structural brain examination including an MP-RAGE sequence at baseline and at least one MRI examination after more than 12 months (Aebi, 2002). The composition of the cohort and further inclusion criteria are described elsewhere (Oberstein et al., 2022). After receiving a detailed description of the study a written consent was provided either by the patients themselves or their authorized legal representatives. The clinical ethics committee of the University of Erlangen-Nuremberg approved the study protocol. For the assessment of amyloidopathy, in contrast to the ADNI cohort, both pathological Aβ42 level and Aβ42/Aβ40 ratio were considered. The N variable of the AT (N) classification was determined in both cohorts using the tTau level alone to avoid circularity, since the regional brain volume was examined as a dependent variable.

### 2.2. Magnetic resonance image (MRI) acquisition and brain volumetry

The protocol for MRI acquisition of the ADNI cohort is described elsewhere (see footnote 1). Structural MRI scans of the ADNI cohort were used in this study from MRI scanner platforms of different manufacturers, provided that all scans from one subject were from one type of device. The MRI acquisition and VBM workflow of the Erlangen cohort have already been described in detail (Oberstein et al., 2022). In short, T1-weighted high-resolution structural MRI were acquired using a 3T MR Scanner (Magnetom Tim Trio 3,0 Tesla, Siemens Healthineers AG, Erlangen, Germany) for brain volumetry of the Erlangen cohort. For processing the T1-weighted structural MRI, we used the VBM workflow for longitudinal models for large changes (e.g., ageing effects) of the Computational Anatomic Toolbox (CAT12 v. 12.8; University Hospital Jena; Jena, Germany) for SPM12^3^ running on MatLab R2021a (Mathworks, Inc.; Natick, Massachusetts, United States). Structural MRI images of the ADNI and the Erlangen cohort were analyzed using the identical workflow. The preprocessed and normalized gray matter maps were used for the group comparisons, and the significant clusters identified by this were characterized using the AAL atlas to determine their anatomical location (Rolls et al., 2020). MarsBar was used to define custom regions of interests (ROIs) based on contrast images from the SPM results of the ADNI data for the Erlangen cohort and to extract GM density from all MRI scans for the ROI analyses (Matthew Brett et al., 2002).

### 2.3. Cerebrospinal fluid-ELISA

The details of the CSF sample collection and analytic processing are described elsewhere^4^ (Shaw et al., 2009). For the AT (N) grouping of the ADNI cohort based on CSF values, we used the archived data set “UPENNBIOMK_MASTER.csv.” A cutoff value of 192 pg./ml was used to determine Aβ42 status in CSF, a cutoff value of 23 pg./ml was used for pTau181 status, and a cutoff value of 93 pg./ml was used for tTau status (Shaw et al., 2009). If multiple CSF values were reported at baseline, we used the median value of these results. The details of CSF sample collection, analytical processing, and cutoff values for the Erlangen cohort are described elsewhere (Oberstein et al., 2022). In short, the cut-offs were calculated by maximizing the respective Youden index of results of the different ELISAs for Aβ42, pTau, and tTau in CSF. A cut-off value of 0.05 was used for the determination of the status of the Aβ42/Aβ40 ratio, a cut-off value of 600 pg./ml (INNOTEST^®^, Innogenetics) or 620 pg./ml (IBL) for Aβ42 status, a cut-off value of 60 pg./ml (INNOTEST^®^, Innogenetics) and 50 pg./ml (INNOTEST^®^, Fujirebio) for pTau181 status, and a cut-off value of 320 (INNOTEST^®^, Fujirebio) or 300 pg./ml (INNOTEST^®^, Innogenetics) for tTau status.

### 2.4. Statistics

Normality was examined using the Shapiro–Wilk test and upon visual inspection of the quantile–quantile-plots. The parametric or nonparametric analyses were applied accordingly. The assumption of homogeneity of variance was assessed with the Levene’s test. Group comparisons were performed with Pearson’s *χ*^2^ for categorical variables and for ordinal or nonnormally distributed interval variables with the Kruskal-Wallis test followed by Dunn multiple comparison test if a significant effect was observed. For normally distributed interval variables, an analysis of variance (ANOVA) or, for groups with inhomogeneous variances, the Brown–Forsythe test was applied, followed by Bonferroni corrected multiple comparison if a significant effect was observed.

Voxel-based morphometry analyses using SPM12 used a flexible factorial analysis of covariance (ANCOVA) to assess changes in GM volumes over two time points (baseline vs. 2-year follow-up or baseline vs. 5-year follow-up) within the four selected ATN groups and differences in longitudinal atrophy between these groups in the ADNI cohort with age as covariate. If no MRI was acquired exactly after 2 or 5 years of follow-up, the next time point was taken, provided this did not change the time interval by more than 12 months. The GM and WM morphological abnormalities are reported after using a family-wise error (FWE) as indicated (*p* ≤ 0.05, *p* ≤ 0.01, or *p* ≤ 0.001). The calculation of the sample size (*n*) of the different groups (i) for the ROI analysis comparing annualized atrophy rates in the Erlangen cohort was computed based on the arithmetic mean of the ADNI cohort (*μ*), the pooled standard deviation (*σ*), and *Z*-values (*Z*) determined as a function of the α- and β-error levels as follows:


$$n_{1}=\left(1+\frac{1}{k}\right)\sigma^{2}{\left(\frac{z_{1-\alpha /2}+z_{1-\beta }}{\mu_{0}-\mu_{1}}\right)}^{2};n_{0}=kn_{1}\text{.}$$

The annualized percent change in an unbiased ROI (uROI), determined by VBM analyses of differences between longitudinal atrophy rates among ATN groups in the ADNI cohort, was computed based on the uROI volumes in MRI scans (*V* in cm^3^) from different time points (t_i_ in months) of individuals in the Erlangen cohort as follows:


$$\Delta u\text{ROI}=\left[(V_{it_{1}}-V_{it_{0}})1200\right]/V_{it_{0}}(t_{1}-t_{0}).$$

We used a one-way analysis of covariance (ANCOVA) to identify main effects of the selected ATN groups on the uROI volume and annualized atrophy rates while controlling for total intracranial volume (TIV), age, time of follow-up and education. Homogeneity of regression slopes was not violated with regard to the dependent variable, as the interaction terms were not statistically significant (*p* > 0.05). The Areas under the curve (AUC) under receiver operating characteristic (ROC) curves were compared in a paired-sample scenario based on the nonparametric methods (DeLong et al., 1988).

Data analysis was performed using the statistical package SPSS (version 28.0; SPSS, Chicago, IL, United States) and MATLAB (version R2021b; The Mathworks Inc., Natick, United States). Quartiles are indicated as follows: 1st quartile = Q1; 3rd quartile = Q3; significance levels are indicated as follows: ^***^p < 0.001; ^**^p < 0.01; ^*^p < 0.05; and ns, not significant.

## 3. Results

### 3.1. Baseline characteristics of the ADNI study population

The voxel-based analysis of 185 non-demented individuals of the ADNI cohort was employed to identify unbiased regions of interest (ROI) based on differential longitudinal atrophy rates between the different AT (N) groups at 2 and 5 years of follow-up. The Aβ42/Aβ40 ratio in CSF has not regularly been assessed in these individuals. In order to indicate this lack of information, subjects from the ADNI cohort are referred to being “Aβ42” instead of “A” positive or negative in the results section, which is why the groups are designated as follows: Aβ42 + T+(N±), Aβ42–T+(N±), Aβ42 + T–(N±) and Aβ42–T–N–. Of the 185 subjects, 115 (62.2%) were male and 70 (37.8%) were female. The mean age at baseline was 75 years with a range from 60 to 89 years. The mean MMSE score at baseline was 28 with a range from 24 to 30. The Aβ42 + T + N ± group represented the largest group with a total number of 60 (32%) subjects, followed by the Aβ42–T–N–group with 50 (27%) subjects, the Aβ42–T + N ± with 43 (23%) subjects, and the Aβ42 + T–N–group with 32 (17%) subjects. There were no subjects with an Aβ42+T–N+ profile in this cohort. The baseline demographics, the MMSE results and the measured CSF-biomarker values are given in detail in Table 1. MMSE, Age, Education at baseline as well as TIV, grey and white matter volume did not differ significantly between the groups (Table 1**)**. Of the 185 subjects included in the study, 180 (69 women) came for follow-up at 2 years, and 99 subjects (40 women) came for follow-up at 5 years. The characteristics of the participants who came for follow-up at 2 and 5 years are shown in Supplementary Table 1.

**Table 1:** Baseline characteristics of the ADNI cohort.

	Aβ42–T–N–		Aβ42–T + N±		Aβ42 + T + N±		Aβ42 + T–N–			Value of *p*
Total (female)	50 (17)		43 (19)		60 (23)		32 (11)		*Chi^2^ (6) = 6.607*	*0.359*
	*M*	SD	*M*	SD	*M*	SD	*M*	SD		
Age [y]	74	7	72	7	74	6	75	5	*F (3, 181) = 0.757*	*0.520*
Education [y]	16	3	17	3	16	3	16	3	*H = 2.788, df = 3*	*0.425*
MMSE	29	1	29	2	28	2	28	2	*H = 3.730, df = 3*	*0.292*
Aβ42 [pg/ml]	242	25	239	27	142	27	147	28	*F (3, 157.227) = 202.925*	*< 0.001*
pTau181 [pg/ml]	18	4	36	9	53	35	18	4	*F (3, 70.021) = 43.577*	*< 0.001*
tTau (pg/ml]	54	14	85	24	108	50	50	18	*F (3, 64.639) = 29.158*	*< 0.001*
TIV [ml]	1,483	159	1,478	149	1,487	140	1,555	152	*F (3, 181) = 2.047*	*0.109*
GM [ml]	545	48	570	57	566	52	558	55	*F (3, 181) = 2.065*	*0.107*
WM [ml]	509	67	485	68	490	58	511	56	*F (3, 181) = 1.889*	*0.133*

### 3.2. Patterns of longitudinal brain atrophy in the Aβ42TN groups of the ADNI cohort

**Figure 1:**
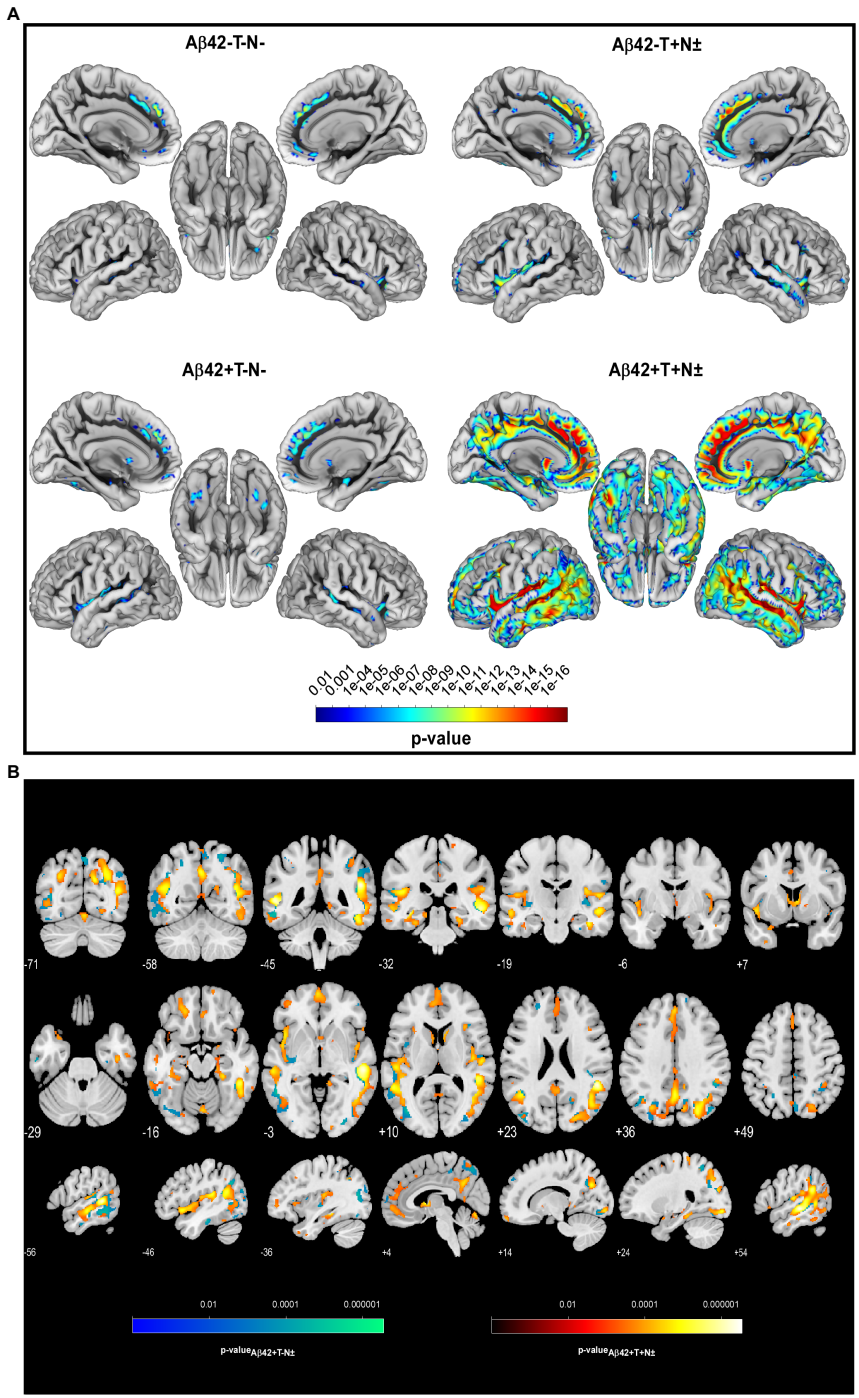
Longitudinal voxel-wise analysis revealed regionally increased atrophy rates in Aβ42–T–N– (*n* = 50), Aβ42–T + N ± (*n* = 43), Aβ42+T–N–. (*n* = 32), and Aβ42 + T + N ± (*n* = 60) non-demented individuals from the ADNI cohort with a mean follow-up of 2 years, *p* < 0.01 (FWE corrected) **(A)**. Slice overlay of the statistical parametric maps illustrates the linear decrease of grey matter (GM) volume that was significantly larger in the Aβ42 + T + N ± (group-red yellow) or the Aβ42 + T–N ± (blue green) compared to the Aβ42–T + N ± group, *p* < 0.01 (FWE corrected) **(B)**. The color bars indicate the value of *p*.

Two years from baseline, the Aβ42 + T + N ± group showed the most widespread longitudinal GM atrophy of all groups (Figure 1; Supplementary Table 2). The volume loss comprised both temporal lobes and reached to parietal and frontal regions including the anterior cingulate gyrus (*p* ≤ 0.01 FWE corrected). The Aβ42 + T–N–group showed a similar pattern of longitudinal GM atrophy as the Aβ42 + T + N ± group, however, in this case the grey matter loss was noticeably to the detriment of the left temporal lobe. In comparison with the Aβ42 + groups, the lowest number of voxels survived the *p* < 0.01 FWE correction in the Aβ42–T + N ± group (Supplementary Table 2). The localization of significant GM loss of the Aβ42–T + N ± group was limited to regions within temporal and insula lobes of both hemispheres. The direct comparison of GM atrophy rates between the Aβ42–T + N ± group and the Aβ42 + T + N ± group showed a significantly greater atrophy rate in regions of the middle temporal gyrus and angular gyrus of the Aβ42 + T + N ± group, *p* ≤ 0.01 (FWE corrected; Figure 1; Table 2). Similarly, the direct comparison of the Aβ42–T + N ± with the Aβ42 + T–N–group showed greater volume loss for the Aβ42 + T–N–group, however, in this case only for clusters in the left hemisphere, *p* ≤ 0.01 (FWE corrected; Figure 1; Table 2). The Aβ42–T + N ± group showed no regions of greater longitudinal GM loss compared to the Aβ42 + T + N ± or the Aβ42 + T–N ± group neither after *p* ≤ 0.01 FWE correction nor without FWE correction (*p* < 0.001, uncorrected). After 5 years, the longitudinal GM loss in the Aβ42–T + N ± group affected more regions within the temporal and insula lobes and was not limited to these anymore but extended to the frontal lobe including the anterior cingulate gyrus (Supplementary Figure 1). The slice overlay of the SPM displaying significantly larger linear volume decline in the Aβ42 + T + N ± compared to the Aβ42–T + N ± group 2 and 5 years from baseline, respectively, indicated only little variation in the maximum intensity projections (MIP; Table 2). The MIP of the SPM 5 years from baseline shifted towards the parietal lobe compared to the MIP of the corresponding SPM 2 years from baseline (Table 2). The clusters surviving the *p* < 0.001 FWE correction of SPM displaying a significant greater linear GM decline of the Aβ42 + T + N ± group compared to the Aβ42–T + N ± group were extracted to serve as a mask for the generation of an unbiased ROI (uROI) for the analysis of longitudinal brain atrophy in the Erlangen cohort (Supplementary Figure 2). The effect size of greater longitudinal atrophy in the uROI of the Aβ42 + T + N ± compared with the Aβ42–T + N ± group was medium after 2 years, Cohen’s *d* = 0.77, and strong, *d* = 1.20, after 5 years. The calculated sample size with a power of 80% and an alpha error of 0.05 was 16 subjects for the Aβ42 + T + N ± and 14 subjects for the Aβ42–T + N ± group based on the effect size for longitudinal atrophy after 5 years.

**Table 2:** Results of two 4 × 2 ANCOVAs with group (Aβ42–T–N–, Aβ42–T + N ±, Aβ42 + T–N ±, Aβ42 + T + N ±) and time point (baseline, follow-up) as independent variables and age as a covariate of no interest.

Brain region	MNI coordinates			Cluster size (Voxels)	% of Cluster	*Z* statistic	Value of *p*
	*x*	*y*	*z*				
Interaction between Aβ42–T + N ± < Aβ42 + T + N ± and t = 0 > t = 2 years							
Left middle temporal lobe	−54	−46.5	4.5	545	97.4	6.40	< 0.001
Left angular gyrus	−45	−55.5	18	545	2.0	5.37	
Outside				545	0.6		
Right middle temporal lobe	52.5	−28.5	−4.5	963	43.2	6.34	< 0.001
Right superior temporal lobe	51	−45	22.5	963	42.6	6.21	
Right supramarginal gyrus	49.5	−36	6	963	5.2	5.80	
Right angular gyrus				963	5.1		
Outside				963	4.0		
Left hippocampus	−34.5	−33	−7.5	25	100.0	5.80	0.002
Left precuneus	−1.5	−61.5	39	123	81.3	5.53	< 0.001
Right precuneus				123	18.7		
Right superior occipital gyrus	28.5	−75	36	194	53.1	5.49	< 0.001
Right middle occipital gyrus				194	46.9		
Right middle temporal lobe	42	−75	15	227	45.8	5.44	< 0.001
Right middle occipital gyrus	43.5	−67.5	18	227	42.7	5.37	< 0.001
Right angular gyrus	42	−57	19.5	227	7.1	5.31	
Outside				227	4.4		
Right inferior temporal lobe	48	−42	−15	58	75.9	5.40	< 0.001
Outside	49.5	−51	−18	58	13.8	4.98	
Right middle temporal lobe				58	5.2		
Right fusiforme gyrus				58	5.2		
Left superior temporal lobe	−43.5	−34.5	16.5	60	91.7	5.39	< 0.001
Left rolandic operculum				60	8.3		
Left middle temporal lobe	−54	−24	−9	47	100.0	5.29	< 0.001
Interaction between Aβ42–T + N ± < Aβ42 + T–N ± and t = 0 > t = 2 years							
Left middle temporal lobe	−52.5	−45	0	225	100.0	5.91	< 0.001
Left middle temporal lobe	−43.5	−57	16.5	28	100.0	5.25	0.003
Interaction between Aβ42–T + N ± < Aβ42 + T + N ± and t = 0 > t = 5 years							
Right angular gyrus	45	−52.5	30	301	89.37	6.02	< 0.001
Right superior temporal lobe				301	4.32		
Outside				301	3.99		
Right middle temporal lobe				301	2.33		
Left middle temporal lobe	−45	−54	15	115	100	5.75	< 0.001
Right middle temporal lobe	51	−36	4	31	93.55	5.28	0.002
Outside				31	6.45		

MNI = Montreal Neurological Institute; FWE = family wise error. Only cluster sizes > 25 voxels were reported, *p* < 0.01 (FWE corrected).

### 3.3. Baseline characteristics of the Erlangen study population

The Erlangen cohort comprised 62 individuals with an A + T + N±, A–T + N ±, or A–T–N–profile of whom 35 (56%) were male and 27 (44%) were female. The mean age was 63 ± 8 years, the median MMSE score was 28 [27; 29], and the mean years of education were 14 ± 4 years (Table 3). The median follow-up period was 48 months with a range from 13 to 104 months with no significant difference in the mean follow up times between the different ATN profiles (Table 3**)**. The baseline characteristics of the Erlangen cohort are given in Table 3. The A + T + N ± group was significantly older than the A–T–N–group (*p* = 0.017, M_Diff_ = 7.199, *95%* CI [1.02; 13.38]). Apart from that, the groups did not differ in terms of age, sex, length of education, and MMSE score at baseline in Bonferroni corrected pairwise comparisons. The A–T + N ± not only showed increased pTau181-levels compared with the A–T–N–group, but also showed significantly increased Aβ40 levels (*p* < 0.001, M_Diff_ = 5720.492, *95%* CI [2121.71; 9319.27]) and tTau levels (*p* < 0.015, M_Diff_ = 120.491, *95%* CI [18.73; −222.26]). In turn, in the A + T + N ± group, significantly lower Aβ42 levels (*p* < 0.001, M_Diff_ = −522.983, *95%* CI [−817.0595; −228.9071]), lower Aβ42/Aβ40 ratio (*p* < 0.001, M_Diff_ = −0.042945, *95%* CI [−0.05082; −0.03507]), and increased pTau181 levels (*p* < 0.001, M_Diff_ = 69.1957, *95%* CI [53.325; 85.067]) were associated with increased tTau levels (*p* < 0.001, M_Diff_ = 382.361, *95%* CI [491.56; 273.16]) compared with the control group.

**Table 3:** Baseline characteristics of the Erlangen cohort.

	A–T–N–		A–T + N±		A + T + N±			Value of *p*
*n* total (female)	26 (14)		19 (4)		17 (8)		*Chi^2^ (2) =* 5.643	0.060
	*M*	SD	*M*	SD	*M*	SD		
Age [years]	61	8	62	9	68	7	*F (2, 59) =* 4.467	0.016
Education [years]	14	3	15	3	13	4	*F (2, 59) =* 1.380	0.259
MMSE	28	2	28	2	27	2	*F (2, 59) =* 1.621	0.207
Follow-up [m]	60	25	52	27	42	23	*F (2, 59) =* 2.590	0.084
GM [ml]	590	61	548	137	537	68	*F (2, 59) =* 1.920	0.156
TIV [ml]	1,469	123	1,526	160	1,415	162	*F (2, 59) =* 2.602	0.083
Aβ1-42	1,201	342	1,472	467	678	204	*F (2, 59) =* 20.560	< 0.001
Aß1-40	12,286	3,527	18,007	3,217	19,969	7,569	*F (2, 59) =* 14.678	< 0.001
Aβ1-42/1–40 Ratio	0,08	0,01	0,07	0,01	0,04	0,01	*F (2, 59) =* 100.435	< 0.001
pTau	38	11	71	9	108	36	*F (2, 59) =* 58.332	< 0.001
tTau	212	58	332	112	594	227	*F (*2, 59*) =* 37.522	< 0.001

### 3.4. Longitudinal unbiased ROI and hippocampal atrophy is significantly larger in the A + T + groups compared to the A–T + and A–T–groups with no difference between the latter

**Figure 2:**
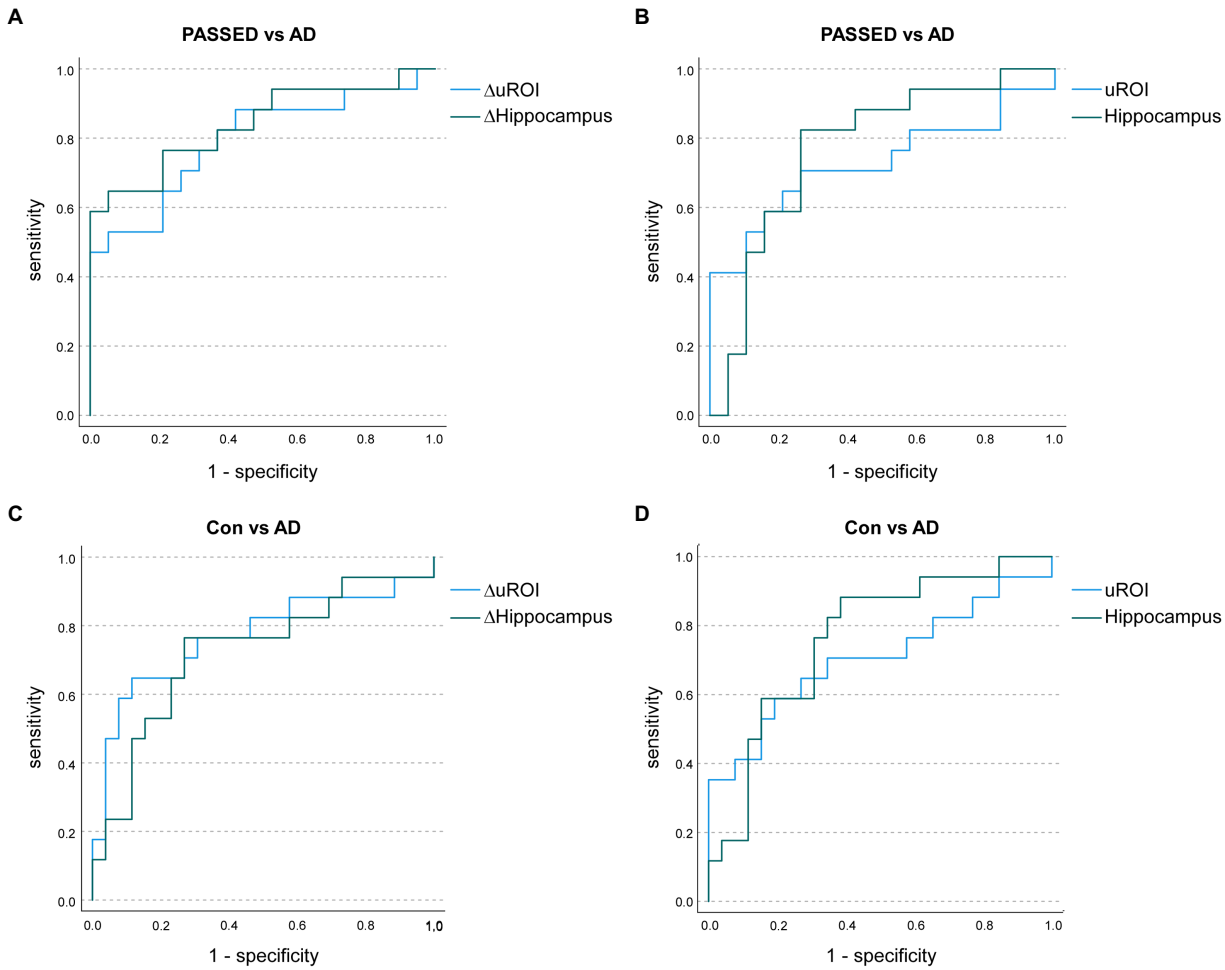
Receiver operating characteristic (ROC) curves illustrate the annualized longitudinal atrophy rates in the unbiased region of interest analysis (ROI; ΔuROI) and the hippocampus (ΔHippocampus) to distinguish A–T + (PASSED) individuals (*n* = 19) from A + T + (AD) nondemented individuals (*n* = 17) from the Erlangen Cohort **(A)**. Area under the curve (AUC) values showed no significant difference between groups. Baseline uROI atrophy and hippocampal atrophy also showed no significant difference between PASSED and AD **(B)**. Similarly, when A–T–N– (biomarker-negative) subjects (*n* = 26) and AD subjects were compared in terms of annualized longitudinal **(C)** or baseline **(D)** atrophy, no significant differences in AUC values were observed.

An ANCOVA with age at baseline, years of education, TIV, and time of follow-up as covariates was conducted to compare the annualized atrophy rate of uROI (ΔuROI) identified in the ADNI cohort between the A + T + N ±, A–T + N ±, and A–T–N–groups of the Erlangen cohort. The groups differed statistically significantly in the ΔuROI, *F* (2, 53) = 124.734, *p* = 0.002, partial *η*^2^ = 0.213. *A priori* contrasts showed statistically significant higher longitudinal atrophy of the unbiased ROI in the A + T + N ± group than in the A–T + N ± group, *M*_Diff_ = −3.951, 95%-CI[−6.133, −1.769], *F*(1, 53) = 13.187, *p* < 0.001, partial *η*^2^ = 0.199 or the A–T–N–group, *M*_Diff_ = −3.101, 95%-CI[−5.144, −1.058], *F*(1, 53) = 9.266, *p* = 0.004, partial *η*^2^ = 0.149. No significant difference was found in comparison between the A–T + N ± and the A–T–N–groups. Unadjusted means and means of the longitudinal atrophy of the uROI adjusted for age, years of education, TIV, and follow-up time in months are given in Supplementary Table 3. No statistically significant difference in the selected ATN groups was found for atrophy at baseline in the brain region of the unbiased ROI or the hippocampi. ΔuROI had an area under the curve (AUC) of 78.8% (*95%* CI = 69.3–97.4) and uROI had an AUC of 72.2% (*95%* CI = 54.1–92.9) to classify nondemented participants between A–T + N±, i.e., PASSED, and A + T + N± (Figure 2). Compared with the AUCs of longitudinal hippocampal atrophy rate and hippocampal atrophy, neither the ΔuROI (*Z* = 0.04, *p* = 0.698) nor the uROI (*Z* = 0.778, *p* = 0.431) in the Erlangen cohort did discriminate better between PASSED and A + T + N ±. Between the biomarker negative control group, A–T–N–, and the A + T + N ± group, there were similar AUCs for the ΔuROI (AUC of 76.9% [*95%* CI = 60.6–93.3]) and the ROI (AUC of 70.1% [*95%* CI = 52.4–87.8]) (Figure 2). Again, there was no significant difference between the ΔuROI (*Z* = −0.699, *p* = 0.485) and ROI (*Z* = 0.555, *p* = 0.579) compared to longitudinal hippocampal atrophy and baseline hippocampal atrophy.

## 4. Discussion

In this study, non-demented individuals with increased CSF-pTau181 levels without amyloidopathy (A–T + N ±) showed no significantly greater longitudinal grey matter atrophy in any brain region compared to controls (A–T–N–) or non-demented AD individuals (A + T + N ±) as assessed by VBM. Similar to the A + T + N ± group, the A–T + N ± group exhibited the most severe longitudinal atrophy rate in the medial temporal lobes and the singular gyrus. These findings are in accordance with previous reports that the atrophy of nondemented individuals with SNAP shows great overlap with the atrophy of nondemented individuals with AD, particularly in the medial temporal lobe, despite possibly different underlying pathologies (Jack et al., 2016; Wisse et al., 2021). However, compared to AD patients, longitudinal atrophy appears to be less pronounced in SNAP patients and only slightly more or indistinguishable from biomarker negative controls (Burnham et al., 2016; Jack et al., 2016; Schreiber et al., 2017; Stocks et al., 2022). In the ADNI cohort investigated in this study, a region with overlap in the angular gyrus of the A–T + N ± group showed significantly less longitudinal atrophy than that in the A + T + N ± group. This was also confirmed in the second cohort of the Erlangen cohort. This is accordance with previous reports, which identified the angular gyrus as a signature region for AD (Dickerson et al., 2017). However, as conducted by ROC Analyses, the longitudinal atrophy of the identified region in the angular gyrus did not discriminate better between A–T + N ± and A + T + N ± individuals than the longitudinal atrophy of the hippocampal volume, which has already been established to measure neurodegeneration (N; Jack et al., 2018b). In summary, the A–T + N ± group exhibited no evidence of pronounced longitudinal brain atrophy in nondemented individuals compared with controls, whereas both groups showed less atrophy in the hippocampus and in the ROI overlapping with the angular gyrus compared with AD individuals. Moreover, in the Erlangen cohort, no pronounced hippocampal atrophy or atrophy of the ROI could be detected cross-sectionally compared with controls. This is in contrast to the reported characteristics of SNAP, in which atrophy of the medial temporal lobe and hypometablosimus in temporal–parietal regions as assessed by 18F-Fludeoxyglucose-PET were considered as criteria for its definition (Jack et al., 2012, 2016, 2018b). In addition, as previously reported by others and us, pTau181 and Aβ are positively associated in the A–T + group, which is why we proposed the term PASSED, a pTau and Aβ surge with subtle cognitive deterioration, for this biomarker constellation (DeLong et al., 1988; Delvenne et al., 2022; Oberstein et al., 2022). The absence of brain atrophy both cross-sectionally and longitudinally, the absence of differences in psychometric trajectories that we have previously reported, but a higher mean age of nondemented individuals in this group compared with biomarker-negative controls may suggest that this is not a dementing disease but rather an ageing-associated condition (Oberstein et al., 2022). Despite the similarity to biomarker negative individuals in terms of course, the A–T + group appears to be a distinct condition in which Aβ peptides are elevated in CSF, but a number of other proteins are decreased in CSF and plasma (Delvenne et al., 2022). The differences between PASSED and SNAP in its original definition, i.e., A–T ± N +, need to be clarified in the future. Possibly pTau alone, unlike tTau, is not indicative of neurodegeneration in terms of brain atrophy. However, considering the strong association between pTau and tTau another possibility seems more likely: For the second cohort, not only Aβ42 level but also Aβ42/Aβ40 ratio was considered in the assessment of amyloidopathy. The Aβ42/Aβ40 ratio appears to detect AD earlier, so more individuals with AD in the first group may have been misclassified as A–T + because of the lower sensitivity of the Aβ42 level alone (Lewczuk et al., 2004, 2017). Considering that brain atrophy in AD typically begins in the mesial temporal lobe (McKhann et al., 2011), the misclassification of individuals with abnormal Aβ42/Aβ40 ratio but normal Aβ42 levels could explain the lack of difference between the longitudinal hippocampal atrophy of the A–T + and A + T + groups in the direct comparison in the first cohort. Therefore, we believe it is essential to measure the Aβ42/Aβ40 ratio for comparison of PASSED and SNAP in future studies. The advantages of machine learning methods, such as the ability to use data from different platforms for disease classification, such as MRI, neuropsychological data, and data from omics platforms, may be useful in future studies to determine whether and to what extent SNAP and PASSED are different pathophysiological conditions.

An important strength of our study is the use of two independent datasets. The difference in results between the two datasets underscores the need to consider the method used to detect amyloidopathy, tauopathy, and neurodegeneration or neuronal damage when evaluating ATN classification. One limitation is that the study population of the Erlangen cohort was not randomly selected from the community and was generally well educated, precluding extrapolation of study results to the general population. Furthermore, subjects in the ADNI cohort were on average significantly older than those in the Erlangen cohort. When interpreting longitudinal atrophy rates in the Erlangen cohort, it should be noted that follow-up intervals differed between subjects. Finally, in this study, the number of A + T–N ± and A–T–N + subjects was too small to draw conclusions about the interaction of these groups with those studied.

In summary, nondemented subjects with elevated pTau levels without amyloidopathy and Aβ surge with subtle cognitive decline (PASSED) did not show a unique longitudinal atrophy pattern compared with nondemented subjects with AD. The lack of a significant difference between atrophy rates in PASSED and controls suggests that elevated pTau181 levels without concomitant amyloidopathy are not indicative of a neurodegenerative disorder.

## Data availability statement

Publicly available datasets were analyzed in this study. This data can be found at: https://adni.loni.usc.edu/data-samples/access-data/.

## Ethics statement

The studies involving human participants were reviewed and approved by Ethik-Kommission der Friedrich-Alexander-Universität Erlangen-Nürnberg, Erlangen, Germany. The patients/participants provided their written informed consent to participate in this study.

## Author contributions

TO designed the study. TO and A-LH analyzed the data, interpreted the results, and drafted the manuscript. A-LH, PO, JU, and E-MS contributed to the data collection, the diagnostic review process, and coordination of the MRI appointments. PS contributed to the oversight of data collection, the diagnostic review process, and reviewing manuscript. AF contributed to the data interpretation and manuscript revision. MS contributed to the MRI data analysis. AD oversaw the MRI data collection and contributed to the manuscript revision. PL contributed to the data analysis, the diagnostic review process, interpretation of results, and manuscript revision. JK oversaw the clinical data collection and contributed to the interpretation of findings, the diagnostic review process, and revision of the manuscript. JM contributed to the study design, oversight of data collection, the diagnostic review process, and manuscript revision, and obtained ethics permission. All authors contributed to the article and approved the submitted version.

## Funding

We received financial support from Deutsche Forschungsgemeinschaft and Friedrich-Alexander-Universität Erlangen-Nürnberg within the funding programme “Open Access Publication Funding”.

Data collection and sharing for this project was funded by the Alzheimer’s Disease Neuroimaging Initiative (ADNI; National Institutes of Health Grant U01 AG024904) and DOD ADNI (Department of Defense award number W81XWH-12-2-0012). ADNI is funded by the National Institute on Aging, the National Institute of Biomedical Imaging and Bioengineering, and through generous contributions from the following: AbbVie, Alzheimer’s Association; Alzheimer’s Drug Discovery Foundation; Araclon Biotech; BioClinica, Inc.; Biogen; Bristol-Myers Squibb Company; CereSpir, Inc.; Cogstate; Eisai Inc.; Elan Pharmaceuticals, Inc.; Eli Lilly and Company; EuroImmun; F. Hoffmann-La Roche Ltd. and its affiliated company Genentech, Inc.; Fujirebio; GE Healthcare; IXICO Ltd.; Janssen Alzheimer Immunotherapy Research & Development, LLC.; Johnson & Johnson Pharmaceutical Research & Development LLC.; Lumosity; Lundbeck; Merck & Co., Inc.; Meson Scale Diagnostics, LLC.; NeuroRx Research; Neurotrack Technologies; Novartis Pharmaceuticals Corporation; Pfizer Inc.; Piramal Imaging; Servier; Takeda Pharmaceutical Company; and Transition Therapeutics. The Canadian Institutes of Health Research is providing funds to support ADNI clinical sites in Canada. Private sector contributions are facilitated by the Foundation for the National Institutes of Health (www.fnih.org). The grantee organization is the Northern California Institute for Research and Education, and the study is coordinated by the Alzheimer’s Therapeutic Research Institute at the University of Southern California. ADNI data are disseminated by the Laboratory for Neuro Imaging at the University of Southern California.

## Conflict of interest

PL received consultation and/or lecture honoraria from IBL International, Fujirebio Europe, AJ Roboscreen, Biogen, and Roche.

The remaining authors that the research was conducted in the absence of any commercial or financial relationships that could be construed as a potential conflict of interest.

## Publisher’s note

All claims expressed in this article are solely those of the authors and do not necessarily represent those of their affiliated organizations, or those of the publisher, the editors and the reviewers. Any product that may be evaluated in this article, or claim that may be made by its manufacturer, is not guaranteed or endorsed by the publisher.

## Footnotes

^1^ www.loni.ucla.edu/ADNI

^2^ www.adni-info.org

^3^ https://www.fil.ion.ucl.ac.uk/spm/

^4^ https://adni.loni.usc.edu/methods/
