# Supplementary material for: PASSED: Brain atrophy in non-demented individuals in a long-term longitudinal study from two independent cohorts
Source: Front Aging Neurosci. 2023 Feb 22;15:1121500. doi: 10.3389/fnagi.2023.1121500 (PMC9992803; doi:10.3389/fnagi.2023.1121500)
Supplement: Supplementary file 1 [file Table_1.docx]

Supplementary Material

**PASSED: Brain Atrophy in non-demented individuals in a long-term longitudinal study from two independent cohorts**

**Supplementary Figures**

**Supplementary Tables**

**Supplementary Table 1 |** Baseline characteristics of participants from the ADNI who had a follow up after 2 and 5 years

|  | Aβ42–T–N– | | | | |  | Aβ42–T+N± | | | | |  | Aβ42+T+N± | | | | |  | Aβ42+T–N– | | | | |
| --- | --- | --- | --- | --- | --- | --- | --- | --- | --- | --- | --- | --- | --- | --- | --- | --- | --- | --- | --- | --- | --- | --- | --- |
|  | FUP 2 years | |  | FUP 5 years | |  | FUP 2 years | |  | FUP 5 years | |  | FUP 2 years | |  | FUP 5 years | |  | FUP 2 years | |  | FUP 5 years | |
|  | 50 (17) | |  | 23 (12) | |  | 40 (17) | |  | 25 (11) | |  | 59 (23) | |  | 36 (12) | |  | 31 (12) | |  | 15 (5) | |
|  | *M* | *SD* |  | *M* | *SD* |  | *M* | *SD* |  | *M* | *SD* |  | *M* | *SD* |  | *M* | *SD* |  | *M* | *SD* |  | *M* | *SD* |
| Age [y] | 74 | 7 |  | 73 | 5 |  | 73 | 7 |  | 72 | 6 |  | 74 | 6 |  | 74 | 6 |  | 74 | 5 |  | 75 | 5 |
| Education [y] | 16 | 3 |  | 15 | 4 |  | 16 | 3 |  | 16 | 3 |  | 16 | 3 |  | 16 | 3 |  | 15 | 4 |  | 15 | 4 |
| MMSE | 29 | 1 |  | 29 | 1 |  | 29 | 1 |  | 29 | 1 |  | 28 | 2 |  | 29 | 1 |  | 28 | 2 |  | 28 | 2 |
| Amyloid beta 42 [pg/ml] | 245 | 22 |  | 242 | 22 |  | 238 | 22 |  | 240 | 23 |  | 138 | 28 |  | 143 | 18 |  | 150 | 26 |  | 157 | 31 |
| pTau181 [pg/ml] | 18 | 3 |  | 17 | 3 |  | 35 | 9 |  | 36 | 10 |  | 57 | 38 |  | 56 | 40 |  | 17 | 4 |  | 18 | 4 |
| tTau (pg/ml] | 55 | 13 |  | 54 | 12 |  | 70 | 22 |  | 71 | 19 |  | 93 | 46 |  | 88 | 33 |  | 50 | 15 |  | 53 | 15 |
| TIV [ml] | 1482 | 158 |  | 1416 | 127 |  | 1471 | 157 |  | 1504 | 138 |  | 1486 | 141 |  | 1505 | 148 |  | 1557 | 157 |  | 1634 | 116 |
| GM [ml] | 545 | 48 |  | 533 | 36 |  | 564 | 56 |  | 578 | 59 |  | 564 | 51 |  | 573 | 51 |  | 558 | 56 |  | 576 | 42 |
| WM [ml] | 502 | 66 |  | 485 | 63 |  | 484 | 66 |  | 495 | 68 |  | 490 | 56 |  | 492 | 62 |  | 488 | 57 |  | 537 | 45 |
| CSF [ml] | 435 | 101 |  | 398 | 79 |  | 424 | 96 |  | 431 | 96 |  | 432 | 95 |  | 439 | 79 |  | 490 | 88 |  | 521 | 64 |
| FUP = follow-up period | | | | | | | | | | | | | | | | | | | | | | | |
|  | | | | | | | | | | | | | | | | | | | | | | | |

**Supplementary Table 2** | Results of two 4×2 ANCOVAs with group (Aβ42-T-N-, Aβ42-T+N±, Aβ42+T-N± , Aβ42+T+N± ) and time point (baseline , follow-up) as independent variables and age as a covariate of no interest. Only cluster sizes >25 voxels were reported, p < 0.001 (FWE corrected)

| Brain region | MNI coordinates | | |  | Cluster size (Voxels | Cluster size (Voxels) | *Z statistic* | *p-Value* |
| --- | --- | --- | --- | --- | --- | --- | --- | --- |
|  | x | y | z |  |  |  |  |  |
| Aβ42-T+N±; t = 0 > t = 2 years |  |  |  |  |  |  |  |  |
| **TPOmid R** | **60** | **-1.5** | **-13.5** |  | **44** | **50** | **5.8** | **< .001** |
| STG R |  |  |  |  | 44 | 43.18 |  |  |
| TPOmid R |  |  |  |  | 44 | 6.82 |  |  |
| **Insula R** | **46.5** | **-4.5** | **3** |  | **27** | **92.59** | **5.7** | **< .001** |
| Rolandic Operculum R |  |  |  |  | 27 | 7.41 |  |  |
| **Cerebelum lobule VI R** | **37.5** | **-64.5** | **-21** |  | **25** | **64** | **5.7** | **< .001** |
| Fusiforme gyrus R |  |  |  |  | 25 | 36 |  |  |
|  |  |  |  |  |  |  |  |  |
| Aβ42-T-N-; t = 0 > t = 2 years |  |  |  |  |  |  |  |  |
| **PHG L** | **-21** | **7.5** | **-24** |  | **47** | **40.43** | **6.2** | **< .001** |
| OFCpost L |  |  |  |  | 47 | 34.04 |  |  |
| Outside |  |  |  |  | 47 | 14.89 |  |  |
| **TPOsup L** |  |  |  |  | 47 | 10.64 |  |  |
| **Insula R** | **48** | **1.5** | **1.5** |  | **89** | **87.64** | **6.1** | **< .001** |
| Rolandic Operculum R |  |  |  |  | 89 | 12.36 |  |  |
| **SFGmed L** | **1.5** | **40.5** | **31.5** |  | **152** | **98.03** | **6.0** | **< .001** |
| ACCsup L |  |  |  |  | 152 | 0.66 |  |  |
| ACCpre R | 0 | 46.5 | 21 |  | 152 | 0.66 | 5.7 | < .001 |
| ACCpre L |  |  |  |  | 152 | 0.66 |  |  |
| **SMA L** | **1.5** | **3** | **49.5** |  | **32** | **65.62** | **5.7** | **< .001** |
| SMA R |  |  |  |  | 32 | 34.38 |  |  |
|  |  |  |  |  |  |  |  |  |
| Aβ42+T-N±; t = 0 > t = 2 years | x | y | z |  |  |  |  |  |
| **MTG L** | **-51** | **-45** | **1.5** |  | **2964** | **67.61** | **7.4** | **< .001** |
| ITG L |  |  |  |  | 2964 | 14.1 |  |  |
| Outside |  |  |  |  | 2964 | 6.48 |  |  |
| STG L | -63 | -24 | -27 |  | 2964 | 3.27 | 6.7 | < .001 |
| Fusiforme gyrus L |  |  |  |  | 2964 | 3.04 |  |  |
| TPOmid L | -55.5 | -1.5 | -37.5 |  | 2964 | 2.97 | 6.7 | < .001 |
| IOG L |  |  |  |  | 2964 | 1.65 |  |  |
| Angular gyrus L |  |  |  |  | 2964 | 0.67 |  |  |
| MOG L |  |  |  |  | 2964 | 0.2 |  |  |
| **Hippocampus L** | **-24** | **-13.5** | **-16.5** |  | **521** | **92.71** | **7.1** | **< .001** |
| Outside |  |  |  |  | 521 | 5.57 |  |  |
| Amygdala L | -33 | -21 | -13.5 |  | 521 | 1.73 | 6.9 | < .001 |
| **Lingual gyrus L** | **-25.5** | **-64.5** | **-6** |  | **699** | **56.51** | **6.8** | **< .001** |
| Fusiforme gyrus L |  |  |  |  | 699 | 38.91 |  |  |
| Cerebelum lobule IV, V L |  |  |  |  | 699 | 2.15 |  |  |
| Cerebelum lobule VI L |  |  |  |  | 699 | 1.14 |  |  |
| PHG L | -21 | -48 | -12 |  | 699 | 1.14 | 6.5 | < .001 |
| Outside |  |  |  |  | 699 | 0.14 |  |  |
| **TPOsup L** | **-21** | **7.5** | **-24** |  | **111** | **40.54** | **6.6** | **< .001** |
| OFCpost L |  |  |  |  | 111 | 23.42 |  |  |
| PHG L |  |  |  |  | 111 | 18.92 |  |  |
| Outside |  |  |  |  | 111 | 17.12 |  |  |
| **Outside** | **-4.5** | **9** | **10.5** |  | **301** | **47.84** | **6.5** | **< .001** |
| Caudate nucleus L |  |  |  |  | 301 | 28.9 |  |  |
| Caudate nucleus R | 4.5 | 9 | 0 |  | 301 | 23.26 | 6.5 | < .001 |
| **STG right** | **60** | **-4.5** | **-10.5** |  | **1194** | **43.72** | **6.4** | **< .001** |
| MTG R |  |  |  |  | 1194 | 38.94 |  |  |
| TPOmid right | 54 | -10.5 | -13.5 |  | 1194 | 16.5 | 6.2 | < .001 |
| TPOsup R |  |  |  |  | 1194 | 0.42 |  |  |
| Outside | 57 | -27 | -3 |  | 1194 | 0.42 | 6.2 | < .001 |
| **IFGorb** | **-48** | **13.5** | **-7.5** |  | **175** | **38.29** | **6.4** | **< .001** |
| TPOsup L |  |  |  |  | 175 | 25.14 |  |  |
| Outside |  |  |  |  | 175 | 17.14 |  |  |
| Insula L |  |  |  |  | 175 | 16.57 |  |  |
| STG L |  |  |  |  | 175 | 2.29 |  |  |
| IFGtri L |  |  |  |  | 175 | 0.57 |  |  |
| **ITG L** | **-43.5** | **-49.5** | **-25.5** |  | **687** | **39.3** | **6.4** | **< .001** |
| Fusiforme gyrus L | -45 | -25.5 | -31.5 |  | 687 | 36.97 | 6.3 | < .001 |
| Cerebelum lobule VI L |  |  |  |  | 687 | 10.04 |  |  |
| Outside |  |  |  |  | 687 | 7.57 |  |  |
| Cerebelum lobule IV, V L | -30 | -31.5 | -27 |  | 687 | 4.08 | 6.1 | < .001 |
| Cerebelum Crus L |  |  |  |  | 687 | 1.75 |  |  |
| PHG L |  |  |  |  | 687 | 0.29 |  |  |
| **Heschl’s gyrus L** | **-46.5** | **-10.5** | **7.5** |  | **73** | **50.68** | **6.4** | **< .001** |
| Rolandic Operculum L |  |  |  |  | 73 | 36.99 |  |  |
| Insula L |  |  |  |  | 73 | 10.96 |  |  |
| STG L |  |  |  |  | 73 | 1.37 |  |  |
| **Precuneus R** | **15** | **-66** | **30** |  | **77** | **80.52** | **6.3** | **< .001** |
| Cuneus R |  |  |  |  | 77 | 19.48 |  |  |
| **SFGmed L** | **0** | **51** | **13.5** |  | **484** | **51.24** | **6.3** | **< .001** |
| ACCpre L | 0 | 43.5 | 31.5 |  | 484 | 23.76 | 6.0 | < .001 |
| ACCpre R |  |  |  |  | 484 | 11.78 |  |  |
| ACCsup L |  |  |  |  | 484 | 6.4 |  |  |
| SFGmed R |  |  |  |  | 484 | 4.13 |  |  |
| Outside |  |  |  |  | 484 | 1.24 |  |  |
| MCC L | 0 | 31.5 | 34.5 |  | 484 | 0.83 | 5.7 | < .001 |
| MCC R |  |  |  |  | 484 | 0.62 |  |  |
| **Insula R** | **49.5** | **4.5** | **-3** |  | **199** | **67.84** | **6.3** | **< .001** |
| Rolandic Operculum R | 43.5 | 19.5 | -3 |  | 199 | 11.56 | 5.7 | < .001 |
| IFGoperc R |  |  |  |  | 199 | 8.04 |  |  |
| STG right |  |  |  |  | 199 | 6.03 |  |  |
| TPOsup R |  |  |  |  | 199 | 4.52 |  |  |
| Outside |  |  |  |  | 199 | 2.01 |  |  |
| **Hippocampus R** | **25.5** | **-13.5** | **-13.5** |  | **256** | **88.28** | **6.3** | **< .001** |
| Outside |  |  |  |  | 256 | 8.59 |  |  |
| Lateral geniculate R |  |  |  |  | 256 | 2.34 |  |  |
| Amygdala R |  |  |  |  | 256 | 0.78 |  |  |
| **MTG R** | **45** | **-60** | **1.5** |  | **178** | **60.11** | **6.3** | **< .001** |
| ITG R | 39 | -60 | -10.5 |  | 178 | 19.66 | 5.8 | < .001 |
| Outside |  |  |  |  | 178 | 7.3 |  |  |
| Fusiforme gyrus R |  |  |  |  | 178 | 6.74 |  |  |
| IOG R |  |  |  |  | 178 | 6.18 |  |  |
| **Hippocampus L** | **-25.5** | **-40.5** | **3** |  | **67** | **83.58** | **6.2** | **< .001** |
| Outside |  |  |  |  | 67 | 16.42 |  |  |
| **ITG R** | **45** | **6** | **-46.5** |  | **145** | **66.9** | **6.2** | **< .001** |
| Outside |  |  |  |  | 145 | 26.21 |  |  |
| TPOmid right | 34.5 | 9 | -45 |  | 145 | 6.9 | 5.8 | < .001 |
| **STG L** | **-55.5** | **-22.5** | **12** |  | **139** | **59.71** | **6.1** | **< .001** |
| Supramarginal gyrus L |  |  |  |  | 139 | 21.58 |  |  |
| Postcentral gyrus L |  |  |  |  | 139 | 14.39 |  |  |
| Rolandic Operculum L |  |  |  |  | 139 | 2.16 |  |  |
| Outside |  |  |  |  | 139 | 2.16 |  |  |
| **MTG L** | **-64.5** | **-52.5** | **-9** |  | **58** | **89.66** | **6.1** | **< .001** |
| ITG L |  |  |  |  | 58 | 10.34 |  |  |
| **Cerebelum lobule IV, V R** | **28.5** | **-31.5** | **-30** |  | **131** | **93.89** | **6.1** | **< .001** |
| Outside | 28.5 | -36 | -40.5 |  | 131 | 3.05 | 5.5 | < .001 |
| Cerebellum lobule X R |  |  |  |  | 131 | 1.53 |  |  |
| Fusiforme gyrus R |  |  |  |  | 131 | 1.53 |  |  |
| **Outside** | **30** | **-13.5** | **-37.5** |  | **140** | **65** | **6.0** | **< .001** |
| Fusiforme gyrus R | 37.5 | -15 | -40.5 |  | 140 | 33.57 | 5.8 | < .001 |
| ITG R |  |  |  |  | 140 | 1.43 |  |  |
| **Fusiforme gyrus L** | **-28.5** | **-12** | **-36** |  | **33** | **100** | **6.0** | **< .001** |
| **SFGorb L** | **0** | **61.5** | **-7.5** |  | **35** | **77.14** | **5.8** | **< .001** |
| SFGorb R |  |  |  |  | 35 | 22.86 |  |  |
| **Fusiforme gyrus R** | **28.5** | **-39** | **-13.5** |  | **27** | **81.48** | **5.8** | **< .001** |
| PHG R |  |  |  |  | 27 | 18.52 |  |  |
|  |  |  |  |  |  |  |  |  |
| Aβ42+T+N±; t = 0 > t = 2 years |  |  |  |  |  |  |  |  |
| **MTG L** | **-43.5** | **16.5** | **-6** |  | **20156** | **24.9** | **> 8.0** | **< .001** |
| ITG L |  |  |  |  | 20156 | 10.49 |  |  |
| Insula L | -45 | 1.5 | -4.5 |  | 20156 | 9.83 | > 8.0 | < .001 |
| Insula L | -43.5 | -12 | 3 |  | 20156 |  | > 8.0 | < .001 |
| STG L |  |  |  |  | 20156 | 9.65 |  |  |
| Fusiforme gyrus L |  |  |  |  | 20156 | 5.71 |  |  |
| Hippocampus L |  |  |  |  | 20156 | 5.51 |  |  |
| Outside |  |  |  |  | 20156 | 5.51 |  |  |
| TPOsup L |  |  |  |  | 20156 | 4.48 |  |  |
| Rolandic Operculum L |  |  |  |  | 20156 | 4.43 |  |  |
| MOG L |  |  |  |  | 20156 | 3.21 |  |  |
| Angular gyrus L |  |  |  |  | 20156 | 2.83 |  |  |
| Heschl’s gyrus L |  |  |  |  | 20156 | 1.9 |  |  |
| Lingual gyrus L |  |  |  |  | 20156 | 1.84 |  |  |
| PHG L |  |  |  |  | 20156 | 1.47 |  |  |
| IFGtri L |  |  |  |  | 20156 | 1.4 |  |  |
| IFGorb |  |  |  |  | 20156 | 1.37 |  |  |
| Cerebelum lobule VI L |  |  |  |  | 20156 | 1.06 |  |  |
| TPOmid L |  |  |  |  | 20156 | 0.94 |  |  |
| IOG L |  |  |  |  | 20156 | 0.69 |  |  |
| OFCpost L |  |  |  |  | 20156 | 0.68 |  |  |
| Cerebelum Crus L |  |  |  |  | 20156 | 0.64 |  |  |
| Amygdala L |  |  |  |  | 20156 | 0.47 |  |  |
| Supramarginal gyrus L |  |  |  |  | 20156 | 0.41 |  |  |
| Postcentral gyrus L |  |  |  |  | 20156 | 0.39 |  |  |
| IFGoperc L |  |  |  |  | 20156 | 0.09 |  |  |
| Olfactory cortex L |  |  |  |  | 20156 | 0.05 |  |  |
| Lateral geniculate L |  |  |  |  | 20156 | 0.02 |  |  |
| Calcarine fissure L |  |  |  |  | 20156 | 0.01 |  |  |
| **MTG R** | **46.5** | **0** | **-1.5** |  | **26924** | **19.63** | **> 8.0** | **< .001** |
| ITG R |  |  |  |  | 26924 | 12.74 |  |  |
| STG right | 54 | -28.5 | -3 |  | 26924 | 10.59 | > 8.0 | < .001 |
| Insula R | 43.5 | -15 | 12 |  | 26924 | 9.37 | > 8.0 | < .001 |
| Fusiforme gyrus R |  |  |  |  | 26924 | 7.74 |  |  |
| Outside |  |  |  |  | 26924 | 5.07 |  |  |
| MOG R |  |  |  |  | 26924 | 4.98 |  |  |
| Rolandic Operculum R |  |  |  |  | 26924 | 3.98 |  |  |
| Hippocampus R |  |  |  |  | 26924 | 3.69 |  |  |
| Angular gyrus R |  |  |  |  | 26924 | 3.52 |  |  |
| SOG R |  |  |  |  | 26924 | 2.59 |  |  |
| PHG R |  |  |  |  | 26924 | 2.35 |  |  |
| TPOmid right |  |  |  |  | 26924 | 2.09 |  |  |
| Lingual gyrus R |  |  |  |  | 26924 | 1.95 |  |  |
| TPOsup R |  |  |  |  | 26924 | 1.48 |  |  |
| Supramarginal gyrus R |  |  |  |  | 26924 | 1.44 |  |  |
| Heschl´s gyrus R |  |  |  |  | 26924 | 0.96 |  |  |
| OFCpost R |  |  |  |  | 26924 | 0.82 |  |  |
| Cerebelum lobule VI R |  |  |  |  | 26924 | 0.69 |  |  |
| IFGtri R |  |  |  |  | 26924 | 0.69 |  |  |
| Amygdala R |  |  |  |  | 26924 | 0.6 |  |  |
| IFGorb R |  |  |  |  | 26924 | 0.58 |  |  |
| SPG R |  |  |  |  | 26924 | 0.55 |  |  |
| Cerebelum Crus R |  |  |  |  | 26924 | 0.53 |  |  |
| IFGoperc R |  |  |  |  | 26924 | 0.45 |  |  |
| IOG R |  |  |  |  | 26924 | 0.24 |  |  |
| Cerebelum lobule IV, V R |  |  |  |  | 26924 | 0.24 |  |  |
| Olfactory cortex R |  |  |  |  | 26924 | 0.16 |  |  |
| Cuneus R |  |  |  |  | 26924 | 0.08 |  |  |
| Cerebellum Crus R |  |  |  |  | 26924 | 0.06 |  |  |
| Postcentral gyrus R |  |  |  |  | 26924 | 0.04 |  |  |
| Lateral geniculate R |  |  |  |  | 26924 | 0.03 |  |  |
| Cerebellum lobule X R |  |  |  |  | 26924 | 0.01 |  |  |
| Cerebellum lobule III R |  |  |  |  | 26924 | 0 |  |  |
| **SFGmed L** | **-4** | **40.5** | **31.5** |  | **17261** | **15.54** | **> 8.0** | **< .001** |
| Precuneus R |  |  |  |  | 17261 | 9.62 |  |  |
| Precuneus L |  |  |  |  | 17261 | 8.24 |  |  |
| MCC L |  |  |  |  | 17261 | 7.73 |  |  |
| MCC R |  |  |  |  | 17261 | 6.88 |  |  |
| Outside |  |  |  |  | 17261 | 5.11 |  |  |
| SFGorb L |  |  |  |  | 17261 | 4.95 |  |  |
| SMA L |  |  |  |  | 17261 | 4.53 |  |  |
| ACCpre L |  |  |  |  | 17261 | 4.32 |  |  |
| SFGorb R |  |  |  |  | 17261 | 3.22 |  |  |
| SFGmed R |  |  |  |  | 17261 | 2.9 |  |  |
| Caudate nucleus L |  |  |  |  | 17261 | 2.68 |  |  |
| SMA R |  |  |  |  | 17261 | 2.65 |  |  |
| ACCpre R |  |  |  |  | 17261 | 2.59 |  |  |
| Caudate nucleus R | 10.5 | 16.5 | 13.5 |  | 17261 | 2.52 | > 8.0 | < .001 |
| PCC L |  |  |  |  | 17261 | 2.03 |  |  |
| ACCsup L |  |  |  |  | 17261 | 1.92 |  |  |
| Cuneus R |  |  |  |  | 17261 | 1.89 |  |  |
| Calcarine fissure R |  |  |  |  | 17261 | 1.72 |  |  |
| Gyrus rectus L |  |  |  |  | 17261 | 1.68 |  |  |
| Cuneus L |  |  |  |  | 17261 | 1.47 |  |  |
| Olfactory cortex L |  |  |  |  | 17261 | 0.86 |  |  |
| ACCsub L |  |  |  |  | 17261 | 0.85 |  |  |
| Calcarine fissure L |  |  |  |  | 17261 | 0.78 |  |  |
| Gyrus rectus R |  |  |  |  | 17261 | 0.62 |  |  |
| ACCsup R |  |  |  |  | 17261 | 0.53 |  |  |
| PCC R |  |  |  |  | 17261 | 0.48 |  |  |
| SOG L |  |  |  |  | 17261 | 0.35 |  |  |
| ACCsub R |  |  |  |  | 17261 | 0.34 |  |  |
| Olfactory cortex R |  |  |  |  | 17261 | 0.29 |  |  |
| Ventral striatum L |  |  |  |  | 17261 | 0.17 |  |  |
| Paracentral lobule R |  |  |  |  | 17261 | 0.16 |  |  |
| Lingual gyrus R |  |  |  |  | 17261 | 0.15 |  |  |
| Paracentral lobule L |  |  |  |  | 17261 | 0.14 |  |  |
| Ventral striatum R |  |  |  |  | 17261 | 0.05 |  |  |
| Lingual gyrus L |  |  |  |  | 17261 | 0.04 |  |  |
| Vermis Lobule IV, V |  |  |  |  | 17261 | 0.01 |  |  |
| **Outside** | **-10.5** | **-27** | **18** |  | **68** | **100** | **7.54** | **< .001** |
| **IFGtri L** | **-37.5** | **7.5** | **31.5** |  | **949** | **56.38** | **7.51** | **< .001** |
| IFGtri L | -45 | 30 | 19.5 |  | 949 |  | 6.51 | < .001 |
| Precentral gyrus L | -49.5 | 12 | 31.5 |  | 949 | 23.92 | 6.56 | < .001 |
| IFGoperc L |  |  |  |  | 949 | 10.75 |  |  |
| MFG L |  |  |  |  | 949 | 8.54 |  |  |
| Outside |  |  |  |  | 949 | 0.42 |  |  |
| **Hippocampus L** | **-9** | **-37.5** | **3** |  | **131** | **25.95** | **7.38** | **< .001** |
| Lingual gyrus L |  |  |  |  | 131 | 23.66 |  |  |
| Outside |  |  |  |  | 131 | 21.37 |  |  |
| Precuneus L |  |  |  |  | 131 | 20.61 |  |  |
| Cerebelum lobule IV, V L |  |  |  |  | 131 | 4.58 |  |  |
| PCC L |  |  |  |  | 131 | 3.82 |  |  |
| **IPG R** | **39** | **-54** | **57** |  | **772** | **61.79** | **7.35** | **< .001** |
| IPG R | 39 | -42 | 45 |  | 772 |  | 7.08 | < .001 |
| IPG R | 31.5 | -48 | 43.5 |  | 772 |  | 6.47 | < .001 |
| SPG R |  |  |  |  | 772 | 21.24 |  |  |
| Outside |  |  |  |  | 772 | 9.2 |  |  |
| Supramarginal gyrus R |  |  |  |  | 772 | 3.89 |  |  |
| Angular gyrus R |  |  |  |  | 772 | 2.85 |  |  |
| Postcentral gyrus R |  |  |  |  | 772 | 1.04 |  |  |
| **MOG L** | **-27** | **-70.5** | **33** |  | **1046** | **65.97** | **7.33** | **< .001** |
| MOG L | -28.5 | -84 | 39 |  | 1046 |  | 6.80 | < .001 |
| MOG L | -27 | -87 | 25.5 |  | 1046 |  | 6.39 | < .001 |
| SPG L |  |  |  |  | 1046 | 14.44 |  |  |
| SOG L |  |  |  |  | 1046 | 12.52 |  |  |
| IPG L |  |  |  |  | 1046 | 6.69 |  |  |
| Outside |  |  |  |  | 1046 | 0.38 |  |  |
| Outside | 12 | -27 | 18 |  | 45 | 100 | 7.20 | < .001 |
| **Fusiforme gyrus L** | **-28.5** | **-12** | **-34.5** |  | **186** | **98.39** | **7.19** | **< .001** |
| Outside |  |  |  |  | 186 | 1.61 |  |  |
| **SFGdor L** | **-28.5** | **64.5** | **-3** |  | **1541** | **71.71** | **7.04** | **< .001** |
| SFGdor L | -28.5 | 60 | 13.5 |  | 1541 |  | 7.02 | < .001 |
| MFG L | -24 | 36 | 43.5 |  | 1541 | 26.28 | 7.00 | < .001 |
| Outside |  |  |  |  | 1541 | 1.88 |  |  |
| OFCant L |  |  |  |  | 1541 | 0.13 |  |  |
| **IFGoperc R** | **42** | **19.5** | **25.5** |  | **496** | **76.41** | **6.85** | **< .001** |
| IFGoperc R | 42 | 10.5 | 31.5 |  | 496 |  | 6.85 | < .001 |
| IFGtri R |  |  |  |  | 496 | 19.15 |  |  |
| Precentral gyrus R |  |  |  |  | 496 | 4.44 |  |  |
| **Lingual gyrus R** | **10.5** | **-36** | **4.5** |  | **110** | **52.73** | **6.78** | **< .001** |
| Outside |  |  |  |  | 110 | 24.55 |  |  |
| Precuneus R |  |  |  |  | 110 | 17.27 |  |  |
| Cerebelum lobule IV, V R |  |  |  |  | 110 | 2.73 |  |  |
| Hippocampus R |  |  |  |  | 110 | 1.82 |  |  |
| PCC R |  |  |  |  | 110 | 0.91 |  |  |
| **SFGdor R** | **31.5** | **64.5** | **-1.5** |  | **134** | **98.51** | **6.71** | **< .001** |
| Outside |  |  |  |  | 134 | 1.49 |  |  |
| **SFGdor R** | **28.5** | **49.5** | **36** |  | **226** | **62.39** | **6.60** | **< .001** |
| MFG R | 28.5 | 43.5 | 43.5 |  | 226 | 37.61 | 6.26 | < .001 |
| **Putamen R** | **22.5** | **7.5** | **4.5** |  | **168** | **76.79** | **6.60** | **< .001** |
| Pallidum R |  |  |  |  | 168 | 21.43 |  |  |
| Outside |  |  |  |  | 168 | 1.79 |  |  |
| **SFGdor R** | **30** | **-3** | **61.5** |  | **333** | **96.1** | **5.70** | **< .001** |
| SFGdor R | 25.5 | 6 | 57 |  | 333 |  | 6.23 | < .001 |
| MFG R | 24 | 15 | 51 |  | 333 | 3.9 | 6.51 | < .001 |
| **OFCant** | **33** | **39** | **-18** |  | **72** | **90.28** | **6.46** | **< .001** |
| IFGorb R |  |  |  |  | 72 | 5.56 |  |  |
| OFCpost R |  |  |  |  | 72 | 4.17 |  |  |
| **IPG L** | **-31.5** | **-58.5** | **40.5** |  | **284** | **95.07** | **6.42** | **< .001** |
| Angular gyrus L | -30 | -51 | 46.5 |  | 284 | 2.82 | 6.21 | < .001 |
| SPG L |  |  |  |  | 284 | 1.76 |  |  |
| Outside |  |  |  |  | 284 | 0.35 |  |  |
| **OFCant L** | **-33** | **37.5** | **-15** |  | **127** | **66.14** | **6.22** | **< .001** |
| OFCpost L |  |  |  |  | 127 | 27.56 |  |  |
| OFClat L |  |  |  |  | 127 | 3.15 |  |  |
| IFGorb | -24 | 31.5 | -16.5 |  | 127 | 3.15 | 6.02 | < .001 |
| **Precentral gyrus L** | **-28.5** | **-4.5** | **63** |  | **59** | **50.85** | **6.10** | **< .001** |
| SFGdor L |  |  |  |  | 59 | 49.15 |  |  |
| **Putamen L** | **-22.5** | **-3** | **10.5** |  | **33** | **100** | **6.09** | **< .001** |
| **MFG L** | **-43.5** | **55.5** | **-3** |  | **85** | **76.47** | **5.92** | **< .001** |
| IFGorb | -49.5 | 45 | -4.5 |  | 85 | 15.29 | 6.00 | < .001 |
| IFGtri L |  |  |  |  | 85 | 8.24 |  |  |
| **SFGdor R** | **33** | **48** | **24** |  | **56** | **51.79** | **5.98** | **< .001** |
| MFG R |  |  |  |  | 56 | 48.21 |  |  |
| **Outside** | **1.5** | **-22.5** | **9** |  | **26** | **100** | **5.97** | **< .001** |
| **MFG L** | **-42** | **48** | **12** |  | **54** | **79.63** | **5.90** | **< .001** |
| IFGtri L |  |  |  |  | 54 | 20.37 |  |  |
| **Supramarginal gyrus R** | **63** | **-46.5** | **31.5** |  | **33** | **93.94** | **5.80** | **< .001** |
| Angular gyrus R |  |  |  |  | 33 | 6.06 |  |  |
| **SFGdor R** | **19.5** | **58.5** | **28.5** |  | **26** | **100** | **5.77** | **< .001** |
| **Lingual gyrus R** | **7.5** | **-70.5** | **-9** |  | **34** | **82.35** | **5.74** | **< .001** |
| Cerebelum lobule VI R |  |  |  |  | 34 | 14.71 |  |  |
| Cerebelum lobule VI R |  |  |  |  | 34 | 2.94 |  |  |

ACCpre = Pregenual anterior cingulate cortex; ACCsub = Subgenual anterior cingulate cortex; ACCsup = Supracallosal anterior cingulate cortex; FWE = family wise error; IFGorb = Inferior frontal gyrus, orbital part; IFGoperc = Inferior frontal gyrus, opercular part; IFGtri = Inferior frontal gyrus, triangular part; IPG = Inferior parietal gyrus; IOG = Inferior occipital gyrus; ITG = Inferior temporal gyrus; L = left; MCC = Middle cingulate cortex; MFG = Middle frontal gyrus; MNI = Montreal Neurological Institute; MOG = Middle occipital gyrus; MTG = Middle temporal gyrus; OFCant = Anterior orbital gyrus; OFClat = Lateral orbital gyrus; OFCpost = Posterior orbital gyrus; PCC = Posterior cingulate cortex; PHG = Parahippocampal gyrus; R = right; SFGdor = Dorsolateral superior frontal gyrus; SFGmed = Medial superior frontal gyrus; SFGorb = Medial orbital superior frontal gyrus; SMA = Supplementary motor area; SPG = Superior parietal gyrus; SOG = Superior occipital gyrus; STG = Superior temporal gyrus; TPOmid = Middle temporal pole;

| **Supplementary Table 3** Unadjusted and adjusted mean values of the unbiased region of interest (uROI) atrophy and annualized atrophy rates (ΔuROI) in the A-T-N-, A-T+N±, and A-T+N± groups. | | | | | | | | | | |
| --- | --- | --- | --- | --- | --- | --- | --- | --- | --- | --- |
|  |  | *M* | | | *SE* | | *95% CI* | | | |
|  |  |  |  |  |  |  | *Lower Bound* | | *Upper Bound* | |
|  |  | *Unadj.* |  | *Adj.* | *Unadj.* | *Adj.* | *Unadj.* | *Adj.* | *Unadj.* | *Adj.* |
| uROI | A–T–N– | 0.40 |  | 0.40 | 0.12 | 0.11 | 0.38 | 0.38 | 0.43 | 0.42 |
|  | A–T+N± | 0.41 |  | 0.40 | 0.14 | 0.12 | 0.38 | 0.38 | 0.44 | 0.43 |
|  | A+T+N± | 0.36 |  | 0.37 | 0.15 | 0.14 | 0.33 | 0.34 | 0.39 | 0.39 |
| ΔuROI | A–T–N– | -1.00 |  | -1.07 | 0.58 | 0.61 | -2.16 | -2.29 | 0.15 | 0.15 |
|  | A–T+N± | -0.35 |  | -0.22 | 0.68 | 0.72 | -1.71 | -1.66 | 1.01 | 1.27 |
|  | A+T+N± | -4.11 |  | -4.17 | 0.70 | 0.77 | -5.51 | -5.72 | -2.71 | -2.62 |
| uROI = unbiased region of interest (uROI); ΔuROI = annualized atrophy rate of the uROI; | | | | | | | | | | |
| Adj. = adjusted for age, education, total intracranial volume (TIV), and time of follow-up; unadj. = unadjusted; Covariates appearing in the model are evaluated at the following values: age at Baseline [y] = 63.44, education [y] = 14.10, TIV [ml] = 1471.7861, follow-up [months] = 52.4032. | | | | | | | | | | |
|  | | | | | | | | | | |
|  | | | | | | | | | | |
|  | | | | | | | | | | |
